# A Clip-with-Line Traction Suture Method for Closing Mucosal Defects after Endoscopic Submucosal Dissection

**DOI:** 10.1155/2021/8817726

**Published:** 2021-03-02

**Authors:** Chong Wang, Yiting Wang, Yuanyuan Li, Sheng Zeng, Youxiang Chen, Guohua Li

**Affiliations:** Department of Gastroenterology, The First Affiliated Hospital of Nanchang University, Jiangxi, China

## Abstract

Endoscopic submucosal dissection (ESD) is a technically difficult endoscopic procedure for treating gastrointestinal diseases. Procedure time is longer, and complications such as mucosal defects, intraoperative perforation, and bleeding occur frequently. Here, to solve these problems, we described the clip-with-line traction suture method that applied and performed for closing mucosal defects after ESD in three representative cases.

## 1. Introduction

ESD has been established as a standard treatment for gastrointestinal diseases nowadays. However, this procedure is technically difficult, time-consuming, and has more challenges and complications than conventional endoscopic mucosal resection (EMR), such as large mucosal defects and big perforations. Thus, a variety of suture methods have been proposed, such as clips, OTSC (Over The Scope Clip System), an Endoloop System, and purse-string suture. Because each method has its own merit and demerit, we developed a new method that using a clip-with-line traction suture to close the mucosal defects after ESD in three representative cases.

## 2. Case Reports

### 2.1. Case One

**Figure 1:**
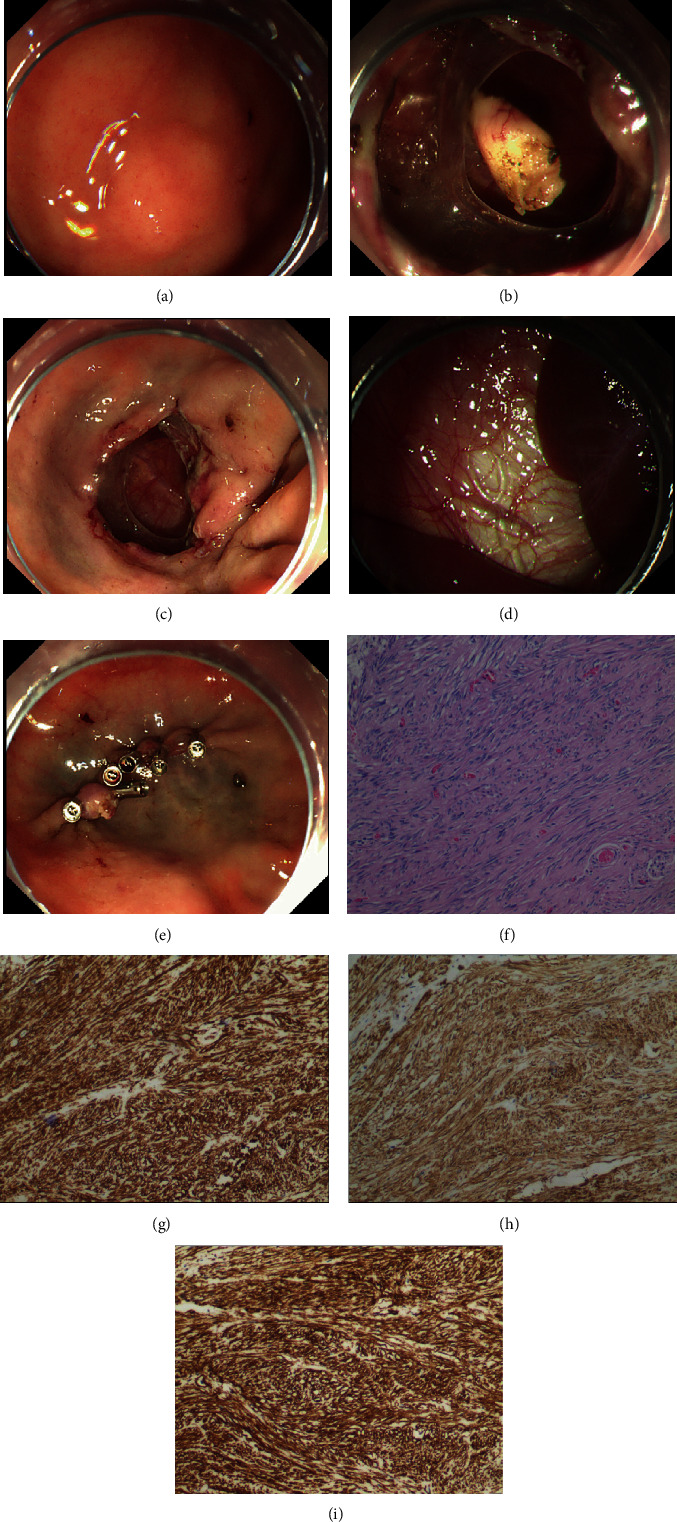
(a) Endoscopy showed that a suspicious protruding lesion was identified in the fundus of stomach. (b) A circumferential incision was produced, and a fundic tumor came into sight. (c) The tumor was resected thoroughly, leaving a big perforation. (d) The visceral organ appeared through the perforation. (e) The clip-with-line traction suture was applied. And the mucosal defect was sutured completely. (f) Haematoxylin and eosin (H&E) staining. Amplification factor: 100-fold. (g) Detection of CD34 protein expression by immunohistochemistry. Amplification factor: 100-fold. (h) Detection of CD117 protein expression by immunohistochemistry. Amplification factor: 100-fold. (i) Detection of DOG1 protein expression by immunohistochemistry. Amplification factor: 100-fold.

A 63-year-old man suffered from a stomachache for about 3 months. Endoscopy diagnosis tends to find a fundic submucosal tumor with a 1.2 cm diameter. After removing the stromal tumor with ESD, there existed a big perforation (Figures 1(a)–1(c)). It was too difficult to close the big perforation with the clips directly. Therefore, we chose the clip-with-line traction method. This method is carried out as follows. A long, 3-0, silk line is tied to the arm part of the clip (*ROCC-D-26-195*, Weichuang, Nanjing, China). The clip applicator device is inserted into the accessory channel of the endoscope. Next, the clip with line is attached to an edge of the target lesion. Then, the lesion is pulled toward outside when the line is pulled very gently, which makes the lesion easier to be closed by clips. After the perforation was closed eventually, the line was removed by APC (Figure 1, Video 1). The procedure was carried out under general anesthesia, the total duration was lasting 15 minutes. The gastric tube was inserted into the stomach for 48 hours. The patient condition was stable with successfully precise free tumor margin of full-thickness resection and discharged five days later without any complications. Haematoxylin and eosin (H&E) showed an atypia neoplastic morphology (Figure 1(g)). Histopathological examination confirmed that representative indicators of gastric stromal tumor such as CD34, CD117, and DOG1 were positively stained (Figures 1(h)–1(i)).

### 2.2. Case Two

A 34-year-old female had a rectum tumor with a 9 mm diameter, which originated from rectum submucosa. After complete resection of the tumor by ESD technique, there was a 2.0 cm diameter big mucosal defect. The wound surface was so large that it was hard to close directly with clips. Consequently, we applied the clip-with-line traction suturing method to close the big mucosal defect. The patient was given the fluid diet for three days and discharged seven days after ESD procedure without any complaints (Table 1).

### 2.3. Case Three

A 66-year-old female developed a 2 cm diameter adenoma, which originated from the rectal mucosa. After complete resection of the adenoma by ESD technique, there was an extensive mucosal defect. We initially performed electrocoagulation on the bleeding mucosa surface. The mucosal defect was so big that it was technically difficult to close with clips. Previous studies suggested that complete closure of the mucosal defect after ESD significantly decreased delayed adverse events (bleeding or perforation). Then, we applied clip-with-line traction suture technique to completely close the mucosal defect. The patient was given the fluid diet for three days and discharged seven days after ESD procedure without any complaints (Table 1).

## 3. Discussion

Our new technique derives originally from the clip-with-line counter traction method reported by Oyama [1] and Jeon et al. [2] It was initially applied to get adequate counter traction and assist in creating a clear vision during ESD. We used firstly the clip-with-line traction method to close mucosal defect. The mucosal defect was closed easily with clips by pulling line, and the line was removed by APC or scissors. The method was previously used to facilitate lesion dissection and removal and has not been used to close mucosal defects during ESD [3, 4].

Compared with clip-with-line method reported by Oyama and Jeon et al., we make improvements in some ways. Firstly, the endoscope need not be withdrawn during operation, which can save time and lower the possible risk of endoscope reinsertion. Secondly, the lines can be mounted on two edges of the big lesion, which make it easier and quicker to close the gap. Furthermore, this novel traction closure can be applied especially to close large mucosal defect and allows endoscopic submucosal dissection to be performed more efficiently and safely. The last but not least, it is also economically feasible; thus, basically, it may be a good choice in poor area. Further advancements are required to evaluate its efficacy and safety through prospective, randomized controlled studies.

## 4. Conclusion

The clip-with-line traction suture method for closing mucosal defects after ESD is quick, safe, efficient, and economically feasible, which can be an optimal choice for poor area.

## Figures and Tables

**Table 1:** General conditions of the patients included in the cases.

General conditions	Case one	Case two	Case three
Sex	Male	Female	Female
Age (years)	63	34	66
Location	Stomach	Rectum	Rectum
Tumor diameter (cm)	1.2	0.9	2.0
Mucosal defect diameter (cm)	2.0	2.0	3.0
Histopathological examination	Gastric stromal tumor	Neuroendocrine tumor (NET)	High-grade intraepithelial neoplasia
Intraoperative duration	Perforation	N.A.	Bleeding
Total duration (mins)	15	19	16
Complications	No.	No.	No.
Postoperative HLOS (days)	5	7	7

HLOS: hospital Length of stay N.A.: not applicable.

## Data Availability

The data used to support the findings of this study are available from the corresponding author upon request.

## Abbreviations

CK: Cytokeratin
ESD: Endoscopic submucosal dissection
EMR: Endoscopic mucosal resection
GIST: Gastrointestinal stromal tumor
H&E: Haematoxylin and eosin
IHC: Immunohistochemistry
OTSC: Over The Scope Clip System
Syn: Synaptophysin.

## Supplementary Materials

Supplementary Materials Video 1: the video clips to an online publically accessible drive (OneDrive), the link as follow, https://1drv.ms/v/s!AmEjpkJRAudkbAAcfhiCIlTQ-zc. After resecting the fundic gastrointestinal stromal tumor with standard ESD procedures, there existed a big perforation. To start with, a long, 3-0, silk line was tied to the arm part of the clip, and the clip with the line was mounted firmly to proximal edge of the fundic wound surface. After that, the line was pulled straightly and tightly with only a small amount of tension so that the distance between wound surface edges could be shortened. Then, the perforation was gradually closed with the clips. In the end, the line was removed by APC device.
