# Supplementary material for: A Clip-with-Line Traction Suture Method for Closing Mucosal Defects after Endoscopic Submucosal Dissection
Source: Gastroenterol Res Pract. 2021 Mar 2;2021:8817726. doi: 10.1155/2021/8817726 (PMC7946448; doi:10.1155/2021/8817726)
Supplement: Supplementary Materials — Video 1: the video clips to an online publically accessible drive (OneDrive), the link as follow, https://1drv.ms/v/s!AmEjpkJRAudkbAAcfhiCIlTQ-zc. After resecting the fundic gastrointestinal stromal tumor with standard ESD procedures, there existed a big perforation. To start with, a long, 3-0, silk line was tied to the arm part of the clip, and the clip with the line was mounted firmly to proximal edge of the fundic wound surface. After that, the line was pulled straightly and tightly with only a small amount of tension so that the distance between wound surface edges could be shortened. Then, the perforation was gradually closed with the clips. In the end, the line was removed by APC device. [file 8817726.f1.doc]

**Video 1:**

The video clips to an online publically accessible drive (OneDrive), the link as follow,

[**https://1drv.ms/v/s!AmEjpkJRAudkbAAcfhiCIlTQ-zc**](https://1drv.ms/v/s!AmEjpkJRAudkbAAcfhiCIlTQ-zc)

After resecting the fundic gastrointestinal stromal tumor with standard ESD procedures, there existed a big perforation. To start with, a long, 3-0, silk line was tied to the arm part of the clip, and the clip with the line was mounted firmly to proximal edge of the fundic wound surface. After that, the line was pulled straightly and tightly with only a small amount of tension so that the distance between wound surface edges could be shortened. Then the perforation was gradually closed with the clips. In the end, the line was removed by APC device.
